# Pyogenic Lumbar Facet Joint Infection with Foot Drop

**DOI:** 10.1155/2021/5544126

**Published:** 2021-04-22

**Authors:** Haruka Maehara, Toshihiro Sano, Yuki Yanagawa, Kyuichi Hashimoto, Nobuaki Tadokoro

**Affiliations:** ^1^Department of Orthopaedic Surgery, Kochi Prefectural Hata Kenmin Hospital, 3-1 Yoshina, Yamana-cho, Sukumo, Kochi 788-0785, Japan; ^2^Department of Orthopaedic Surgery, Susaki Kuroshio Hospital, 4–30, Midorimachi, Susaki, Kochi 785-0036, Japan; ^3^Department of Orthopaedic Surgery, Kochi Medical School, 185-1 Kohasu, Oko-cho, Nankoku, Kochi 783-8505, Japan

## Abstract

Pyogenic facet joint infection (PFJI) is a relatively rare spinal infection. Clinical suspicion of this condition is a key for diagnosis. We report a case of PFJI which required decompression surgery for severe neurological dysfunction. The patient was a 44-year-old woman who had a previous history of orthotic therapy for idiopathic scoliosis. The patient was admitted to our hospital with a history of two days of high fever and severe low back pain. There was no neurologic deficit, and blood tests revealed high levels of inflammatory markers. There was a slight amount of fluid that had collected at L4/5 facet joint in lumbar MRI. She was admitted for examination and treatment of fever of unknown origin and low back pain. Antibiotic treatment started the day after hospitalization since the first report of the blood culture taken upon admission tested positive to gram-positive cocci. As low back pain and fever persisted, an MRI was taken again on the fifth day of hospitalization. Repeated MRI showed fluid extension from the left facet joint to paravertebral muscles and epidural space. She was diagnosed with PFJI, and facet joint puncture was performed. At this time, it became clear that she had foot drop on the right, the contralateral side of the PFJI. She underwent irrigation, debridement, and partial laminectomy. Methicillin-sensitive Staphylococcus aureus (MSSA) was detected in blood cultures at the time of hospitalization, in the puncture fluid and tissue collected during surgery. The patient recovered completely from foot drop after the operation and a three-month course of antibiotics. As the imaging findings may be inadequate in the early stages of onset and PFJI potentially causes neurologic deficit such as foot drop, neurological findings need to be carefully observed even after hospitalization and one should reexamine the MRI if symptoms or clinical findings did not improve or were aggravated.

## 1. Introduction

Pyogenic facet joint infection (PFJI) is a relatively rare spinal infection [1], which is reported to account for 4% of spinal infections overall. Compared with pyogenic spondylodiscitis, diagnostic information on PFJI is still insufficient due to its rarity. We report a case of PFJI which required decompression surgery for severe neurological dysfunction.

## 2. Case Presentation

**Figure 1:**
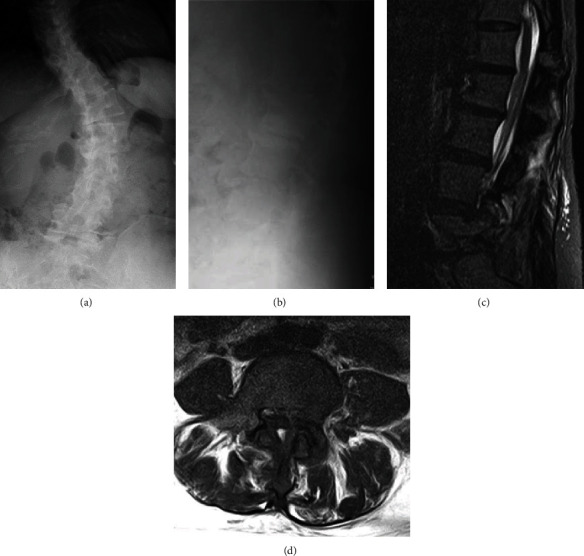
Images of administration on the 5th day of hospitalization. X-ray (a, b) sagittal T2 short tau inversion recovery (T2-STIR) MRI (c), axial L4/5 T2-weighted MRI (d).

A 44-year-old female presented at our emergency department with chief complaints of severe back pain and fever for the past two days. She had a previous history of orthotic therapy for idiopathic scoliosis and no immunosuppressive disease. Her body temperature was 41 degrees, and there was tenderness at the spinous processes of the lower level of the lumbar spine and no neurologic deficit. The white blood cell (WBC) count was 9,250 cells/*μ*L, and C-reactive protein (CRP) was elevated to 13 mg/dL. Although scoliosis was observed on X-ray, no clear bone or joint destruction was confirmed. MRI showed a slight amount of fluid had collected at the left L4/5 facet joint; these findings are considered to be associated with degenerative changes (Figure 1). She was admitted for examination and treatment of fever of unknown origin and low back pain.

**Figure 2:**
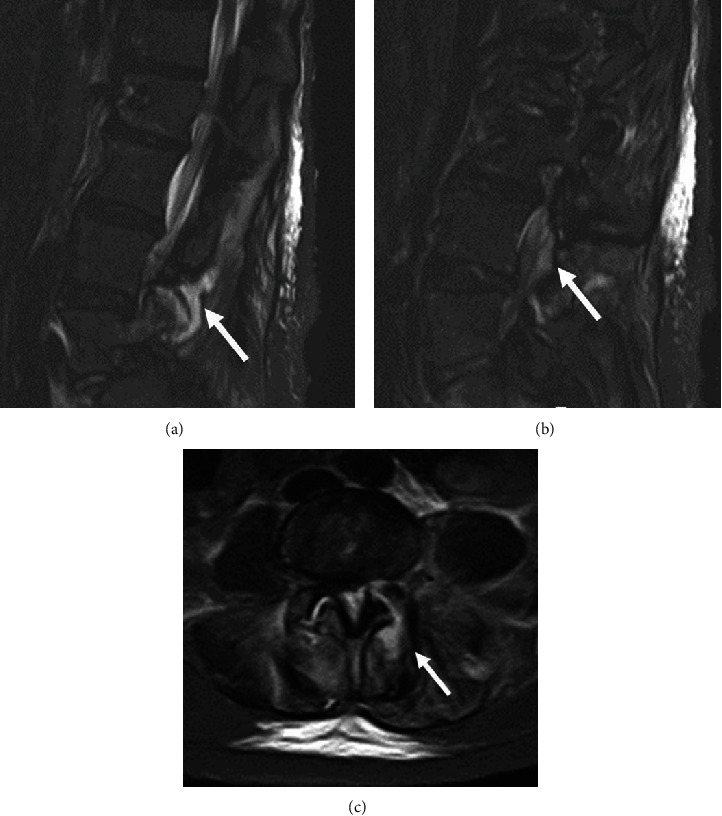
MRI images on the 5th day of hospitalization. Sagittal view (a and b) and axial view at L4/5 (c) of T2-STIR MRI showing an abscess from the facet joint to the paravertebral muscle (a, c) and epidural abscess (arrows).

The first report of the blood culture taken upon admission tested positive to gram-positive cocci, and the patient was started on an intravenous course of sulbactam ampicillin (3 g every 6 hours) and vancomycin (1 g every 8 hours) as the empiric treatment of bacteremia on the second day of hospitalization. Methicillin-sensitive Staphylococcus aureus (MSSA) was isolated in the blood culture on the fourth day after admission, which was sensitive to cephazolin, levofloxacin, and many other antibiotic drugs. And the patient was put on an intravenous antibiotic course of cephazolin (2 g every 8 hours) and oral rifampicin (450 mg every day) for the MSSA bacteremia. On the fifth day after admission, the CRP level was 11 mg/dL, low back pain persisted, and fever was still approximately 38 degrees. Therefore, we took another MRI. The repeated MRI revealed continuous fluid from the facet joint to paravertebral muscles and epidural space (Figure 2). Other fever origins such as bacterial pneumonia and urinary tract infection were negative, and therefore, she was diagnosed with PFJI.

**Figure 3:**
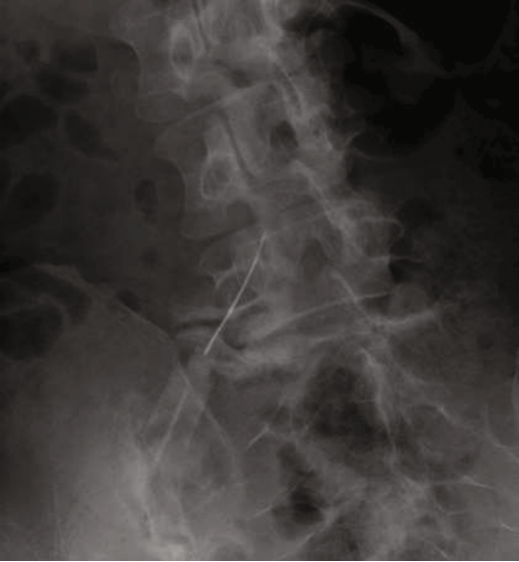
X-ray during left L4/5 facet puncture.

We performed a diagnostic facet joint puncture on the left L4/5 (Figure 3) on the 8th day; the culture tests detected MSSA and were negative for calcium pyrophosphate and uric acid crystals. Thus, we confirmed that it was indeed a pyogenic facet joint infection.

In response to the antibiotic treatment, the CRP level decreased and pain tended to improve, but we observed the right foot drop and MMT3 level muscle weakness in the iliopsoas and quadriceps the day after the puncture. As the atypical findings of lumbar radiculopathy coincided in this case; weakness in the contralateral side of the affected facet joint, and no leg sensory dysfunction including pain and numbness, we searched for other causes of paralysis such as cerebral infarction. However, none were clear, so we performed L4/5 and 5/S1 partial laminectomy and left L4/5 facet joint capsulotomy and irrigation debridement on the 11th day of admission. The epidural space and the left L4/5 facet joint were filled with inflammatory granulomatous tissue.

**Figure 4:**
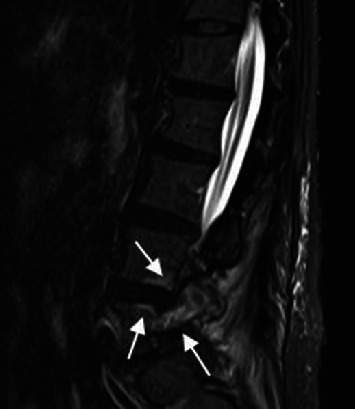
MRI image at two weeks after surgery. Sagittal view of T2-STIR MRI showing bone edema of the left L5 pedicle and L4 and 5 vertebral bodies around the pedicle (arrows).

After the operation, the patient was continued on a course of antibiotics, CRP gradually decreased, and CRP became negative a month after hospitalization. Two weeks after surgery, we took a follow-up MRI, which showed signal changes at the left L5 pedicle and L4 and 5 vertebral bodies around the pedicle, which suggested osteomyelitis (Figure 4). Therefore, we changed the course of antibiotics to oral levofloxacin and rifampicin that has good penetration into the bone and joint [2], and the patient was discharged from our hospital. Muscle weakness in the right lower limbs, including the foot drop, also improved and reached MMT5 levels three months after the admission. Inflammatory changes in the paravertebral muscles and fluid retention in the facet joints had almost disappeared, and bone edema had diminished. Therefore, we decided to discontinue the antibiotic treatment at this stage. The patient took antibiotics for a total of three months. At the one-year follow-up, there was no recurrence of either low back pain, lower limb muscle weakness, or elevated CRP, and no significant instability was observed on the lumbar dynamic X-ray.

## 3. Discussion

Pyogenic arthritis of the facet joint was first reported in 1987 by Halpin and Gibson [3]. It is considered to be a relatively rare disease [1]. However, PFJI was reported to account for 20% of pyogenic spinal infections in 2006 [3]. Compared with pyogenic spondylodiscitis involving disc and vertebral body complex, the facet joints damaged by PFJI are small. Thus, clinical suspicion of this condition is important in order to arrive at a diagnosis.

Symptoms are mainly severe low back pain not relieved by rest and fever. About 40% of patients have some kind of radiculopathy, but severe cases may involve paralysis or cauda equina syndrome, which account for approximately 10% of all cases [1, 4, 5]. The patient in our study also showed typical clinical symptoms of low back pain and fever. Although neurological deficit was not evident upon admission, a foot drop on the contralateral side developed after admission. The reported diverse neurological presentation including the peculiar neurological deficit of this patient might be explained by the varying degree of the abscess formation and the preexisting spinal stenosis. It has been reported that 32% of pyogenic vertebral osteomyelitis patients with severe neurological deficit experienced a deterioration of preexisting neurologic signs or the appearance of a new motor weakness after hospitalization, with a median time to exacerbation of 11 days [6]. As the present case also showed a new neurological deficit, it is necessary to carefully observe the neurological findings after hospitalization, as is the case with pyogenic spondylitis.

MRI is most effective in confirming the diagnosis of PFJI at an early stage [7], and a case has been reported in which effusion of a facet joint and edematous changes of the paravertebral muscle were observed two days after onset [8]. In this case, we can find MRI changes of the facet joint at two days after the onset of symptoms. However, it may be difficult to distinguish it from degenerative findings because there were fewer inflammatory findings in the tissue around the facet joints and the paravertebral muscle abscess and epidural abscess were unclear. Therefore, despite the typical clinical symptoms, the diagnosis was not made on the day of admission. Similarly, there was a case report in which a definitive diagnosis was not made at the first visit, and MRI was reexamined after one month when the symptoms persisted or relapsed [5]. Therefore, we recommend MRI reexamination if symptoms persist or relapse [9].

Paraspinal and/or epidural extension is seen in 81% of cases [4], and in this case as well, it was found to spread to the epidural and paravertebral muscles. Staphylococcus aureus was the most common etiologic organism, and it has been reported to account for approximately 80% of all cases [1, 4]. In our case, MSSA was also detected in blood cultures, facet joint fluid, and infectious tissues collected during surgery.

The focus of the treatment is antibiotic administration and drainage, and surgical treatment is mainly performed on severe neurologic dysfunction, like in this case [4]. The patient was treated with an intravenous course of cefazoline and oral rifampicin as a treatment for MSSA bacteremia and PFJI. The rationale behind this antibiotic treatment is a systematic review from 2014, in which the use of rifampicin as an adjunctive agent in treatment may improve outcomes of Staphylococcus aureus bacteremia [10]. However, in recent years, there have been some reports that the use of rifampicin as an adjunctive agent for Staphylococcus aureus bacteremia does not improve prognosis [11, 12], and therefore, it is necessary to carefully observe the side effects when it is used. No side effects were observed in this case. Although the optimal duration of antibiotic treatment for PFJI has not been defined, intravenous administration for two to four weeks or more followed by oral administration for a total of eight weeks or more is recommended [4, 5]. In this case, osteomyelitis became apparent during the course, and the antibiotic treatment lasted 3 months, because MRI showed a signal change resolution with the maintenance of negative CRP. The patient was treated with levofloxacin and rifampicin as the oral antibiotic drugs for PJFI and osteomyelitis. The combined use of oral levofloxacin and rifampicin is described in the IDSA guidelines for the treatment of vertebral osteomyelitis, and the drugs penetrated into the bone and joint effectively [2, 13].

## 4. Conclusion

We experienced a case of PFJI that required surgery due to foot drop. We need to take PFJI into account when we suspect spinal infection disease. Since the imaging findings may be inadequate in the early stages of onset and PFJI potentially causes neurologic deficit such as foot drop, it was considered necessary to follow-up carefully and reexamine the MRI if symptoms or clinical findings did not improve or became aggravated.

## Data Availability

All the data collected for the work have been reflected in the manuscript.
